# Dendritic Cells as Danger-Recognizing Biosensors

**DOI:** 10.3390/s90906730

**Published:** 2009-08-27

**Authors:** Mia Gi, Wooseok Im, Seokmann Hong

**Affiliations:** Department of Bioscience and Biotechnology, College of Life Science, Sejong University, Seoul, 143-747, Korea; E-Mails: gimia@sejong.ac.kr (M.G.); spon13@naver.com (W.I.)

**Keywords:** dendritic cells, toll-like receptors, pathogen-associated molecular patterns, Interleukin 12, cytokine reporter mouse model

## Abstract

Dendritic cells (DCs) are antigen presenting cells that are characterized by a potent capacity to initiate immune responses. DCs comprise several subsets with distinct phenotypes. After sensing any danger(s) to the host via their innate immune receptors such as Toll-like receptors, DCs become mature and subsequently present antigens to CD4^+^ T cells. Since DCs possess the intrinsic capacity to polarize CD4^+^ helper cells, it is critical to understand the immunological roles of DCs for clinical applications. Here, we review the different DC subsets, their danger-sensing receptors and immunological functions. Furthermore, the cytokine reporter mouse model for studying DC activation is introduced.

## 1. Introduction

Our body is consistently faced with dangerous microorganisms such as bacteria, viruses and fungi. Despite being exposed to a hazardous environment, how can we maintain a healthy body? To protect ourselves from infectious organisms, the immune system plays an important role in fighting against these invaders. To do its job, our immune system has two different weapons: innate immunity and adaptive immunity. While innate immune responses are critical as a first line of defense against pathogens, adaptive immune responses are induced during infection to generate antigen-specific immune responses [1]. For generation of T cell-dependent adaptive immune responses, innate immune responses must be initiated by antigen presenting cells (APCs) such as dendritic cells (DCs). APCs take up antigens and subsequently process them for loading onto major histocompatibility complex (MHC) class II molecules to present to CD4^+^ T cells [2]. DCs, macrophages, and B cells function as professional APCs. Among these, DCs are the most potent APCs that can initiate adaptive immune responses. DCs play critical roles in determining the direction of T cell-mediated immune responses, which consequently influence B cell immune responses such as isotypes of antibodies.

**Figure 1.**
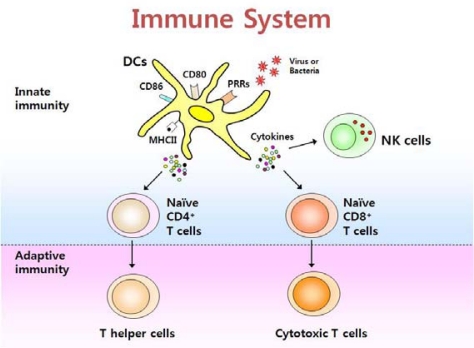
The immune system consists of innate and adaptive immunity. Dendritic cells (DCs) are a crucial element of the immune system, bridging innate and adaptive immunity. Upon stimulation with PAMPs, DCs secrete cytokines that activate natural killer (NK) cells and also help to differentiate CD4^+^ and CD8^+^ T cells.

Innate immune responses initiate from recognition of pathogen-associated signatures such as lipopolysaccharide (LPS). Such pathogen-associated molecules include proteins, DNA and RNA that are unique to the pathogen and do not exist in the host. These signatures are called pathogen-associated molecular patterns (PAMPs). PAMPs include viral dsRNA, bacterial LPS, bacterial lipopeptide, viral and bacterial CpG DNA, and bacterial flagellin [3]. DCs can be activated after recognizing PAMPs through their innate immune receptors. Activated DCs also function as antigen presenting cells by providing pathogen-derived antigens to naïve CD4^+^ and CD8^+^ T cells, by which adaptive immune responses are induced (Figure 1).

In many disease models, DCs play important roles in mediating disease progress or protecting against disease. For this reason, investigation of DCs’ immunological functions provides an understanding of the mechanisms of disease development. Here, we describe the immunological functions of DCs in the immune system and the innate receptors of DCs for recognition of danger signals, and we also introduce the cytokine reporter mouse model to monitor DC activation.

## 2. Dendritic Cells

**Figure 2.**
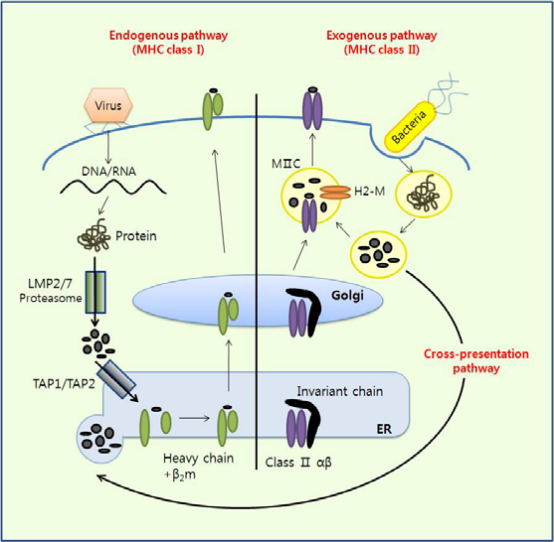
Antigen presentation pathways. The mode of antigen entry into cells and the site of antigen processing determine whether antigenic peptides associate with MHC class I molecules in the endoplasmic reticulum (ER) or with MHC class II molecules in endocytic compartments. However, exogenous antigens can be presented by MHC class I molecules via a process called cross-presentation in some APCs such as CD8^+^ cDCs.

DCs present antigens to naïve CD4^+^ T cells by taking up antigens derived from pathogens. Immature DCs become mature when they recognize PAMPs released from pathogens. During this process, DCs upregulate antigen presenting molecules such as MHC class I and II. MHC class I molecules are designed to present antigenic peptides from intracellular pathogens such as viruses, while MHC class II molecules are designed to present antigenic peptides from extracellular pathogens such as bacteria (Figure 2). Naïve CD4^+^ T cells are primed upon recognition of antigens presented by DCs and begin to differentiate into effector cells [4]. In addition, DCs can cross-present extracellular antigens to CD8^+^ T cells, which play a key role in mediating anti-tumor immunity [5].

For full activation of CD4^+^ T cells by DCs, signals through co-stimulatory molecules such as B7/CD28 are required, in addition to T cell receptor (TCR) signals. During the maturation process of DCs, co-stimulatory molecules on DCs are induced by recognizing PAMPs. If DCs sense non-pathogenic antigens such as self-antigens, they present antigens in an immature status so that T cells recognize antigens loaded onto MHC molecules in the absence of co-stimulatory signals. Without co-stimulatory signals, T cells become anergic even if they bind to MHC/antigen through TCR.

Thus, anergy induced in T cells in response to self-antigens is one mechanism used to achieve peripheral tolerance for suppression of unwanted autoimmune responses. DCs have not only the ability to present antigens to T cells but also the capacity to regulate the micro-environment. CD4 helper T cells are classified into three groups: Th1, Th2, and Th17. While Th1 cells mediate cell-mediated immunity, Th2 cells regulate humoral immunity. Th17 cells, a recently defined subset of helper T cells, can induce inflammatory responses [6]. In addition to these helper T cells, naïve CD4^+^ T cells can differentiate into regulatory T cells depending on the cytokines in the micro-environment. Therefore, DCs control immune responses by either induction or suppression of immune responses. In the next section, we will discuss in detail how CD4^+^ T cells differentiate depending on the stimuli provided by the DCs.

### 2.1. DC Subsets

The DC population consists of heterogeneous groups of cells. All of the components of the DC population are not yet clearly defined. The appearance of new types of DCs makes DC classification more complex and confounding. In general, all DCs in mice express the integrin CD11c, and DC subsets are further defined based on the expression of the myeloid marker CD11b.

Here, we will describe the classifications of the mouse DC populations. Based on recent classifications of DC subsets [7], DCs in mice can be grouped into four subsets : Langerhans cells (LCs), interstitial DCs (IDCs), conventional DCs (cDCs), and plasmacytoid DCs (pDCs) (Table 1). LCs, as a subset of DCs residing at the epithelial surface of skin, respond to pathogens infiltrating through the skin. After their maturation by pathogen-related molecules, LCs migrate to local lymph nodes where they present antigens to Ag-specific T cells. IDCs are one type of DCs that exist in all peripheral tissue except epidermal skin. Like LCs, IDCs can function as APCs. However, IDCs can be distinguished from LCs based on their ability to migrate. Unlike LCs, IDCs can migrate to draining lymph node (DLN) even in the steady state [8]. Two types of DCs reside in lymphoid tissue such as the spleen: CD8α^+^ cDCs and CD8α^−^ cDCs. In a steady state, CD8α^+^ cDCs are primarily located in the T cell zone, while CD8α^−^ cDCs exist in the marginal zone placed at the edge of the sinuses in which the blood flows through lymphoid areas. The difference between CD8α^+^ and CD8α^−^ cDCs is that CD8^+^ cDCs are specialized in cross-presentation of antigens whereas CD8α^−^ cDCs are good at presenting exogenous antigens to CD4^+^ T cells [9]. CD8α^+^ DCs were claimed to be specialized in the induction of anti-viral CD8^+^ T cell responses, in part because of their cross-presenting abilities, whereas CD8α^−^ DCs were proposed to be primarily involved in CD4^+^ T cell immunity, particularly during bacterial infections [10]. pDCs play a pivotal role in defending against viral infection by producing type I interferons (IFNs) which are very effective in inhibiting viral replication [11]. In addition, pDCs can present antigens to CD4^+^ T cells like cDCs. Emerging evidence supports that each subset of DCs has a specialized function in controlling immune responses. Thus, it is necessary to study the immunological roles of the DC subsets to better understand how the immune system is regulated upon encounter of dangers. Since DCs exist in very small numbers (about 1% of immune cells in lymphoid organs), many researchers use in vitro generated DCs by incubating bone marrow (BM) cells with DC growth factors such as fms-like tyrosine kinase 3 ligand (Flt3L). For example, culturing BM cells with Flt3L generates pDCs as well as cDCs [12].

Recently, a new type of DC, called a natural killer dendritic cell (NKDC) was discovered. NKDCs are phenotypically defined by their coexpression of CD11c and NK1.1 [13]. It has been reported that NKDCs show not only cytolytic activity, a characteristic of NK cells, but also can present antigens to T cells like APCs [14,15]. Following tumor cell killing, NKDCs can up-take and present tumor antigens to tumor-specific T cells. These properties make NKDCs very important immune cells in the field of cancer immunology because NKDCs may kill their target and directly cross-present resultant antigens to specific T cells. Although a human counterpart of murine NKDCs has not been identified yet, killer DCs with cytolytic activity have been reported in humans. Whether these killer DCs are *bona fide* NKDCs or not is still under debate [16].

### 2.2. Recognition of Danger Signals by DCs

Danger signals threatening the host can be classified into two groups: exogenous and endogenous signals. While exogenous danger signals induce immune responses through recognition of PAMPs expressed on pathogenic microorganisms, endogenous signals are extracellularly released from normal cells when damaged by chemical or physical insults, resulting in the induction of immune responses. To put these danger molecules together, the new term “damage-associated molecular patterns (DAMPs)” was coined. Upon recognition of DAMPs, DCs induce inflammatory responses and regulate immune responses [3,17].

#### 2.2.1. Pathogen Associated Molecular Patterns and Pattern Recognition Receptors

Aside from DCs, other cells such as epithelial and endothelial cells can recognize PAMPs. The cells stimulated with PAMPs produce a large amount of inflammatory cytokines, which lead to inflammatory responses and also recruit DCs and macrophages by secreting chemokines. PAMPs are detected by innate immune receptors called pattern recognition receptors (PRRs) [18]. Toll-like receptors (TLRs) are one of the PRR families and are expressed either on the cell surface or inside the endosomes [19]. TLRs are mammalian proteins homologous to Toll proteins that were originally identified in fruit fly *Drosophila*. To date, 13 TLR proteins in mice and 10 TLRs in humans have been identified (see Table 2). Among these TLRs, TLR2 can dimerize with TLR1 or TLR6, forming either TLR1/TLR2 or TLR2/TLR6 heterodimers. TLR1/TLR2 recognizes triacylated lipopeptides of bacteria, whereas TLR2/6 recognizes diacylated lipopeptides from mycoplasma. TLR3 and TLR7 recognize viral RNA, TLR3 recognizes dsRNA, and TLR7 recognizes ssRNA. TLR5 recognizes bacterial flagellin. In addition, TLR9 recognizes CpG DNA from either bacteria or viruses.

Most TLRs are expressed in DCs. Upon stimulation by TLR signaling, DCs can induce up-regulation of both MHC molecules and co-stimulatory molecules, and at the same time can produce cytokines such as interleukin (IL)-12. Depending on the subset of DCs, the expression profiles of TLR molecules can vary. In humans, for example, pDCs express TLR1, 6, 7, and 9, whereas cDCs express TLR 1, 2, 3, 5, 6, and 8 [7]. In contrast, most DC subsets express TLRs in mice. As one common factor between human and murine DCs, pDCs are very effective in anti-viral immunity by producing type 1 IFNs via TLR9 or TLR7-mediated activation [20,21]. However, the relationships between any specialized functions of other DC subsets and their TLR expression patterns are not well understood.

The types of cytokines produced by DCs are determined based on the subset of DC and types of PAMPs that they recognize. DCs play critical roles in T helper cell differentiation since naïve CD4^+^ T cells start to differentiate upon recognition of antigens presented by DCs and the polarization of T helper cells into either Th1 or Th2 cells is primarily dependent on the types of cytokines that DCs produce. A lot of evidence supports the regulatory roles of DCs in T helper cell differentiation. For example, differentiation of Th1 cells can be induced by DC-derived IL-12 upon either recognition of CpG via TLR9 or detection of LPS via TLR4. In addition, IFNα secreted by pDCs upon recognition of viral DNA via TLR7 can induce differentiation of naïve CD4^+^ T cells into Th1 cells [22,23]. Although there is still debate as to what type of TLR signaling induces Th2 differentiation, some reports have demonstrated that activation of DCs through TLR2 induced Th2 differentiation [24]. Recently, it has been reported that IL-23 secreted by DCs facilitates differentiation of naïve CD4^+^ T cells into Th17 cells, which are a new type of IL-17-producing T helper cells involved in the pathogenesis of autoimmune diseases such as experimental autoimmune encephalomyelitis (EAE) [25]. In contrast to their roles in the induction of T helper cells, DCs can also induce regulatory T (Treg) cells that have immunosuppressive functions [26]. DCs can produce immunosuppressive cytokines such as transforming growth factor (TGF) β, a key cytokine for the differentiation of Treg cells in vitro and in vivo. DCs can also produce another immunosuppressive cytokine, IL-10, known to induce type I regulatory T (Tr1) cells. Both Treg and Tr1 cells can directly suppress the functions of pathogenic effector T cells [27,28]. Therefore, DCs can control the balance of the immune system by both initiating and suppressing immune responses. Such control of the immune system is dependent on the danger signals that DCs recognize, and the types of cytokines produced by DCs ultimately determine the outcome of immune responses.

Recently, two other PRR families other than TLRs have been identified: NOD-like proteins (NLRs) and RIG-like helicases (RLHs). In contrast to the TLRs that are expressed as transmembrane receptors and recognize PAMPs from extracellular microbes, NLRs and RLHs reside in the cytoplasm and sense PAMPs from intracellular microbes [29]. Whereas NLRs are known to recognize mesodiaminopimelic acid or muramyl dipeptide derived from bacterial peptidoglycan, RLHs sense viral dsRNA like TLR3, but they recognize dsRNA in a TLR3-independent manner [30–32]. However, the immunological roles that these two PRR families play in DCs are not clear.

#### 2.2.2. Alarmins and Their Receptors

In addition to immune responses against invading pathogens, the immune system in the host can react by producing alarmins in response to physical or chemical damages, including burns, cold, chemical insult, radiation, and oxidants. Alarmins are endogenous molecules that function to alert the host to dangers [3]. Alarmin proteins include high-mobility group box 1 (HMGB1, nuclear protein), heat shock protein (HSP, chaperons), uric acid, S100 (calcium-binding protein), and hepatoma-derived growth factor (HDGF, neuron protein). Similar to PAMPs, alarmins function as danger signaling molecules. However, alarmins are different from PAMPs in that they exist as endogenous forms and are released upon necrosis but not apoptosis. Alarmins are recognized by TLR, IL-1R, or receptor of advanced glycation endoproducts (RAGE). Inflammation is induced by signaling through alarmins, which activate the NF-κb signaling pathway. RAGE functions as a receptor for multiple alarmins such as S100, amyloid peptide, and HMGB1. Through RAGE, DCs and macrophages are stimulated to induce inflammatory immune responses [33]. Upon stimulation with alarmins, DCs become mature and induce activation of T cells. For example, DCs activated through HMGB1 up-regulate MHC class II and co-stimulatory molecules such as B7 [34]. On the other hand, DCs produce IL-12 and IFNγ when they are stimulated with uric acid [35]. Considering all of these reports together, alarmins play an important role in non-pathogen-mediated diseases (e.g., cancer, autoimmune diseases) by informing the host of dangers caused by non-infectious agents. In the case of HMGB1, this alarmin is involved in many non-pathogen-mediated diseases including sepsis [36], arthritis [37], atherosclerosis [38], systemic lupus erythematosus (SLE) [39], cancer [40], and hepatitis [41].

### 2.3. Immunological Roles of DCs in Various Diseases

#### 2.3.1. Infectious Diseases

**Figure 3.**
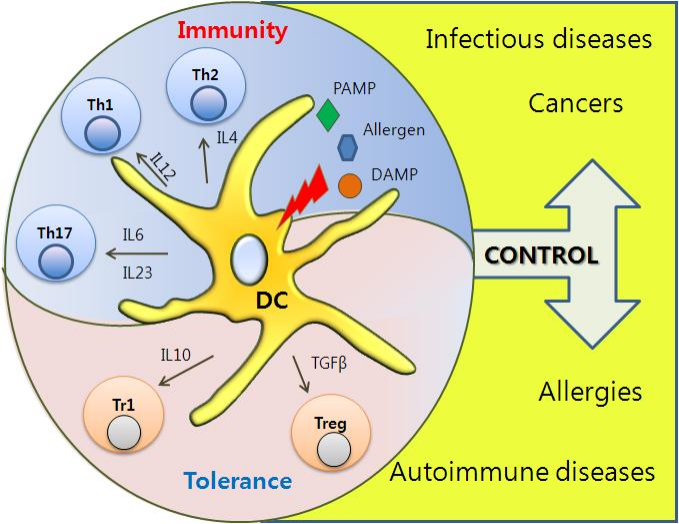
Immunological Roles of DCs. PRR-induced DCs can instruct distinct T helper cell differentiation depending on cytokines, which play a key role in discriminating between immunity and tolerance.

DCs orchestrate the innate and adaptive immune responses and play a central role in properly interpretating signals that maintain tolerance versus those that should be decoded to trigger immune responses against pathogens. PRRs are abundantly expressed by DCs, and DC activation by PAMPs leads to their maturation. PRR-induced DC maturation can instruct distinct programs of T helper cell differentiation depending on the type of polarizing cytokines involved (e.g., IL-12 for Th1, IL-4 for Th2, IL-10 for Tr1, IL-6 for Th17, and TGFβ for Treg) (Figure 3). In addition, TLR triggering plays a key role in discriminating between tolerance and immunity by regulating antigen processing after phagocytosis at the level of phagosome maturation [42]. Therefore, coordination between PRR signals and DC maturation should be tightly controlled in order to maximize immunity yet maintain tolerance. Although triggering of TLR plays a key role in DC maturation through increased expression of co-stimulatory molecules and differential secretion of stimulatory cytokines, the signaling pathways that encode these biological effects are poorly understood.

**Figure 4.**
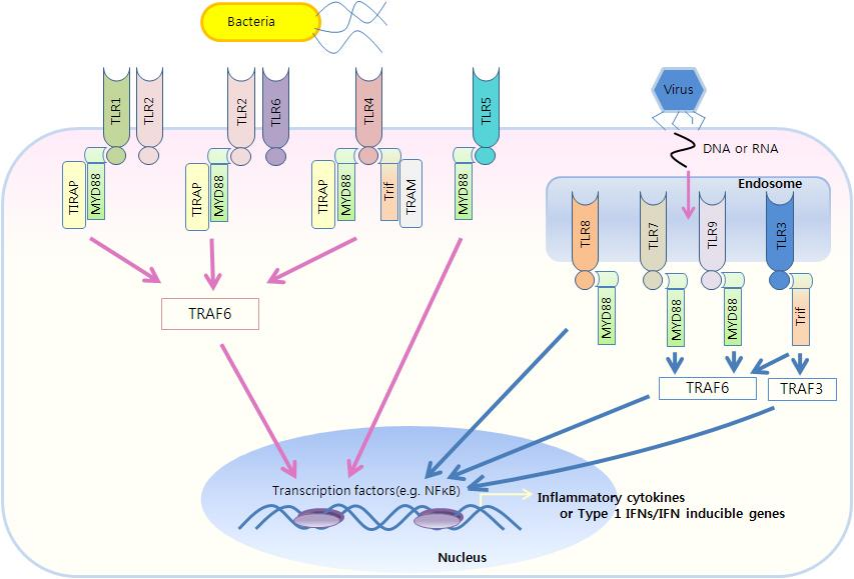
TLR-mediated immune responses. TLR1/2 and TLR2/6 utilize MyD88 and TIRAP as essential adaptors. TLR3 utilizes Trif. TLR4 utilizes four adaptors, including MyD88, TIRAP, Trif, and TRAM. TLR7/8, TLR9 and TLR5 use only MyD88. The MyD88-dependent pathway controls inflammatory responses, while Trif mainly mediates type I IFN responses.

Recent evidence suggests that cytokine expression in DCs is controlled by differential association of tumor necrosis factor (TNF) receptor-associated factor (TRAF) to Toll/IL-1 receptor (TIR)-containing adaptors such as MyD88 and TIR domain containing adaptor protein (TIRAP) (Figure 4) [43]. For example, TRAF3 is essential for the induction of type I IFNs and IL-10, whereas TRAF6 appears to induce IL-6 and TNF [44]. In addition, transcription factors are involved in cytokine induction by DCs. Upon DC stimulation with TLR2 ligands such as yeast zymosan, JNK and p38 activation was correlated with strong IL-12 production, whereas ERK stimulation induced c-Fos, which acted to suppress IL-12 and upregulate IL-10.

Upon loading with microbial antigen and adoptive transfer, DCs are able to induce immunity to infections. For example, the adoptive transfer of DCs pulsed with inactivated human immunodeficiency virus (HIV)-1 into humanized mice could induce HIV-specific CD4^+^ and cytotoxic T lymphocyte (CTL) immune responses, leading to anti-viral immunity [45,46]. On the other hand, in vivo depletion of DCs increased the susceptibility to viral infection [47]. DC-derived IL-12 plays an important role in the induction of resistance to *Leishmania major*. Through experiments using Ag-pulsed LCs from IL-12-deficient mice, it has been shown that the inability to release IL-12 completely abrogates the capacity of LCs to mediate protection against leishmaniasis [48]. In addition, it has recently been reported that DCs in the lung are capable of phagocytosing *Cryptococcus neoformans* in vivo and presenting antigens to *C. neoformans*-specific T cells ex vivo [49]. *C. neoformans*, an opportunistic fungal pathogen, predominantly causes disease in immunocompromised patients such as individuals with AIDS. Although microbial infections normally induce the maturation of DCs, some microbes such as mycobacteria [50] can block DC maturation and also hepatitis C virus modulates DCs to induce Th2 rather than Th1 immune responses [51]. Thus, it is critical to regulate the immune function of DCs for the control of infectious diseases.

#### 2.3.2. Cancer

DCs play important roles in anti-tumor immunity. However, many cancers have evolved strategies to evade immune surveillance. For example, oncogenic proteins expressed by cancers induce up-regulation of STAT3 in DCs, which induce IL-10 and TGFβ expression, leading to deletion of T cells along with anergy induction of T cells by down-regulation of MHC class II molecules and co-stimulatory molecules [52]. It has recently been reported that inhibition of STAT3 in DCs can reverse anti-tumor immunity [53]. Since cancer does not express a “signature of danger signal” that stimulates the maturation and activation of DCs, DCs may not recognize the existence of cancer cells. Using such a phenomenon, many studies have been performed to test whether activation of DCs with adjuvants containing danger signals can reverse the inhibition of immune responses against cancers. For example, during chemotherapy or radiotherapy against cancers, alarmins are normally secreted from cancer cells. These alarmins can activate DCs, facilitating the cross-presentation of antigens derived from cancer cells and enabling production of cytokines such as IL-12 with anti-tumor activity. IL-12 subsequently induces the activation of NK cells and also contributes to the differentiation of CTLs [54]. In addition, activation of DCs with adjuvants containing TLR3 ligands can induce anti-tumor immune responses [55]. Recently, mature DCs loaded with cancer-derived antigens have been used for cancer immunotherapy [56].

#### 2.3.3. Autoimmune Diseases

Autoimmune diseases caused by autoreactive T cells or autoantibody-producing B cells can be initiated when self-antigens are presented by DCs. In addition, DCs in an autoimmune-prone condition can aggravate inflammatory immune responses by secreting IFNα/β and TNFα upon stimulation with necrotic cells caused by autoreactive T cells. TNFα plays a critical role in the pathogenesis of rheumatoid arthritis and psoriasis, and DCs are the primary source of TNFα [57]. Treatment of patients with TNFα-blocking therapeutic agents dramatically improved symptoms of arthritis and psoriasis. SLE is an autoimmune disease, characterized by the breakdown of immune tolerance by nuclear components and often gets worse by viral infection. Recently, it has been reported that pDCs are involved in the pathogenesis of SLE by secreting IFNα/β [58]. pDCs, the main producer of IFNα/β, induce activation of autoreactive T cells and the production of autoreactive antibodies by making immature DCs become mature DCs [59]. It is now believed that pDCs mediate IFNα/β production by signaling through TLR7 and TLR9, which recognize viral nucleic acid, or via DNA-binding proteins such as HMGB1 and RAGE [11,60].

#### 2.3.4. Allergies

Although inhaled allergens are taken up by DCs and presented to T cells, tolerance is induced in healthy individuals. However, in allergic disease conditions, immune tolerance against inhaled allergens becomes broken, resulting in the presentation of allergens by DCs to T cells. Upon recognition of allergens, T cells differentiate toward Th2 cells, which give aid to B cells and produce allergen-specific immunoglobulin (Ig) E. Skin epithelial cells directly or indirectly influence DCs to induce allergic immune responses. Skin epithelial cells help DCs to sample allergens infiltrated into skin and also regulate cytokine production of DCs to help differentiation of naïve CD4^+^ T cells into Th2 cells. Thymic stromal lymphopoietin or granulocyte macrophage colony stimulating factor (GM-CSF), produced by epithelial cells, has been known to induce Th2 polarization [61,62]. In addition, it has been recently reported that inhaled allergens with enzymatic activity act on DCs to induce Th2 differentiation [62].

## 3. Research on DC Activation through Cytokine Reporter Mice

Since DCs initiate adaptive immune responses, research on functions of DCs can provide basic and fundamental information that will be useful for developing therapeutics for a variety of diseases, including cancers. Of particular interest, manipulation of DC activation is important to regulate the adaptive immune responses toward a desired direction. Here, we will introduce a mouse model that has been developed to monitor immune cells producing IL-12, which play a critical role in Th1 and CTL differentiation.

### 3.1. Cytokine Reporter Mouse Model

Cytokine IL-12 plays critical roles in linking innate and adaptive immune responses [63]. This cytokine is a heterodimeric cytokine, sharing a p40 subunit with IL-23. Although IL-12 p40 is mainly produced in DCs and macrophages after stimulation with TLR ligands, methods to monitor the cells that produce IL-12 p40 in vivo were previously limited.

**Figure 5.**
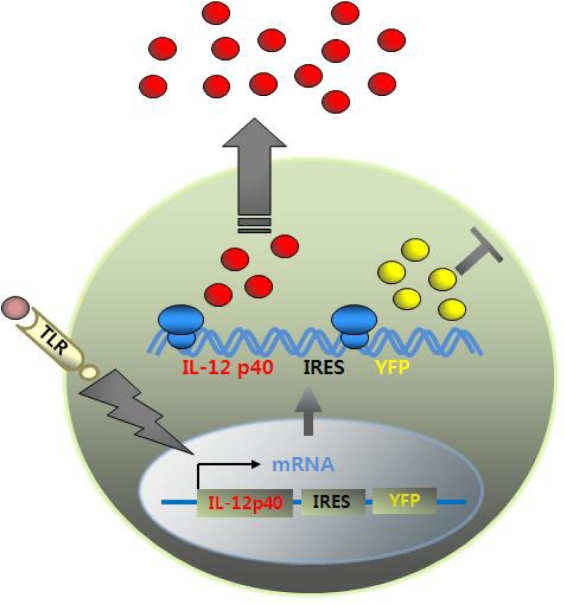
How the cytokine reporter system works. YFP gene is linked to IL-12 p40 gene via IRES element. When DCs are stimulated with TLR ligands, induction of IL-12 p40 gene generates bicistronic transcript encoding both IL-12 p40 and YFP gene. While IL-12 p40 proteins are secreted extracellulary, YFPs remain in the cytosol, marking cells fluorescent.

Recently, an in vivo mouse model that can track p40-expressing cells with fluorescent reporters has been established [64,65]. This IL-12 p40 knockin mouse model was generated to incorporate into mice a reporter cassette that consists of a yellow fluorescent marker gene linked to a viral internal ribosomal entry site (IRES) immediately after the stop codon located in exon 8 of genomic IL-12 p40. Thus, when IL-12-producing immune cells such as DCs and macrophages are stimulated with TLR ligands, induction of the IL-12 p40 gene generates a bicistronic transcript encoding both IL-12 p40 and the yellow fluorescent protein (YFP) gene. Bicistronic transcripts are translated into IL-12 p40 proteins that are subsequently secreted. YFPs remain in the cytosol, consequently marking cells fluorescent (Figure 5).

**Figure 6.**
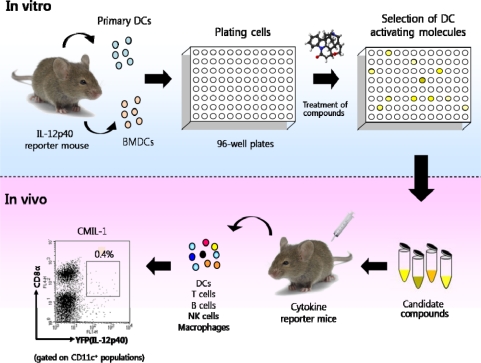
The protocol for screening and characterizing molecules with DC-stimulating activities. In brief, DCs from IL-12 p40 reporter mice are incubated with testing compounds and subsequently YFP-inducing compounds are selected by flow cytometry or fluorescence microscopy. Various cytokine reporter models can be used to examine the candidate compounds for their immunostimulatory effects in vivo. One FACS datum indicates that a subset of DCs can induce IL-12 p40 upon in vivo administration of CMIL-1 which is one of the compounds selected by this procedure (adapted from Ref [64] with permission from The Korean Society for Molecular and Cellular Biology).

Such a system will be used as biosensors allowing us to screen molecules that stimulate DCs. Figure 6 shows the experimental protocol for sorting out molecules with immunostimulatory activities. In brief, as a first step, DCs are isolated by CD11c magnetic-activated cell sorting (MACS) or cultured with DC growth factors (e.g., GM-CSF or Flt3L) from a cytokine reporter system such as IL-12 p40 reporter mice. Next, the purified DCs are overlaid on microwell plates and are subsequently treated with various molecules including herbal extracts. Candidate molecules inducing IL-12 p40 gene expression can be identified with a flow cytometer or a fluorescence imager. Lastly, in vivo administration of the selected molecules into cytokine reporter mice enables us to monitor their physiological immune activities.

**Figure 7.**
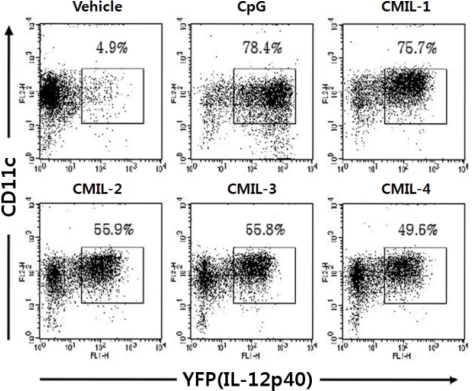
Identification of four bio-compounds inducing IL-12 p40 expression via the cytokine reporter system. Flt3L-cultured BMDCs were stimulated with vehicle, CpG (5 μg/ml), CMIL-1, -2, -3 or -4. Sixteen hours later, IL-12 p40 expression was analyzed by flow cytometry for YFP expression Reprinted from Ref [64] with permission from The Korean Society for Molecular and Cellular Biology.

**Figure 8.**
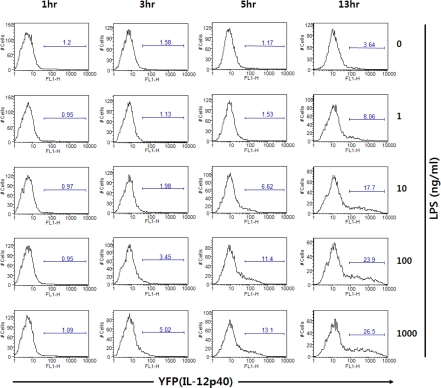
Dose-dependency and kinetics of IL-12 p40 induction by BMDCs upon stimulation with LPS. Flt3L-cultured BMDCs from IL-12 p40 reporter mice were incubated with LPS and analyzed for YFP by flow cytometry.

DCs from the cytokine IL-12 p40 reporter system show the characteristics required for biosensor. Considering the criteria such as selectivity, sensitivity, and kinetics, cytokine reporter-derived DCs can fulfill these requirements as biosensors. When it comes to selectivity, cytokine reporter DCs faithfully perform their jobs to selectively screen the molecules to induce IL-12 p40 gene expression. For example, in the experimental setting for screening IL-12 p40-inducing compounds from more than six hundreds of herbal extracts, only 4 compounds were picked up, which indicates that this system shows remarkable selectivity (Figure 7) [64]. Both sensitivity and kinetics of DCs as biosensors can be investigated by measuring the level of fluorescent proteins expressed in DCs. Figure 8 shows the experimental data measuring sensitivity and kinetics.

Figure 8 shows that DCs can express different levels of fluorescent proteins responding to TLR4 ligand LPS in a dose-dependent manner. In addition to the sensitivity, the kinetics of DC activation can be investigated by using cytokine reporter-derived DCs. In Figure 8, the rate of DC activation in response to LPS can be measured by YFP expression. Challenge of GM-CSF/IL-4-cultured bone marrow-derived DCs (BMDCs) in vitro with LPS resulted in increasing numbers of YFP^+^ cells beginning at about 5 hrs after stimulation. Taken together, DCs derived from the cytokine reporter system can be utilized as biosensors for screening cytokine-inducing molecules.

**Figure 9.**
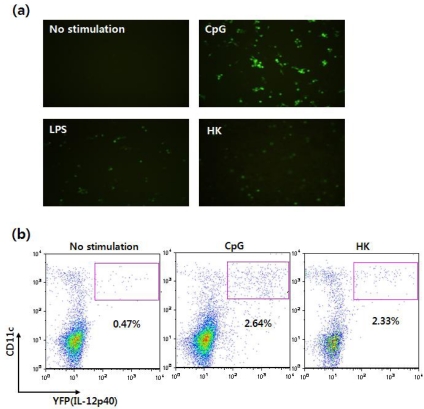
Visualization of IL-12 induction by DCs derived from cytokine reporter mice. **(a)** Flt3L-cultured BMDCs were stimulated with vehicle, CpG, LPS, or heat-killed tumor cells (HK) for 16 hrs, and YFP-expressing cells were observed using fluorescence microscopy. **(b)** Splenocytes were stimulated with vehicle, CpG or HK. Sixteen hours later, IL-12 p40 expression was analyzed by flow cytometry and YFP expression.

Using this reporter system, IL-12 p40-expressing immune cells can be visualized without intracellular cytokine antibody staining, and the cells can be monitored in situ in real time. In fact, when Flt3L-cultured BMDCs were stimulated with CpG or LPS, YFP-expressing cells could be observed with fluorescent microscopy as well as flow cytometry (Figure 9).

There are several advantages of the cytokine reporter system. First, cytokine producing cells can be observed without intracellular cytokine antibody staining, which requires a cell fixation step. Second, viable cytokine producing cells can be isolated with a FACS sorter because fluorescent proteins such as YFPs are not toxic to the cells. Recently, by using IL-12 p40 reporter mice, Reinhardt *et al*. demonstrated that in vitro activated DCs that expressed IL-12 p40 migrated to DLNs and promoted Th1 differentiation more efficiently than DCs that did not express p40 [65] .

Taken together, this cytokine reporter mouse model should prove invaluable, not only in understanding how activation of innate immune cells such as DCs regulates adaptive immune responses including infectious diseases and tumor immunity but also in evaluating novel compounds that can act as adjuvants to promote more effective immune responses [64,66]. We have recently used the IL-12 p40 reporter system to demonstrate that poly-γ-glutamic acid, an edible and safe exopolymer generated by microorganisms such as *Bacillus subtilis*, can modulate T helper cell differentiation by activation of DCs [67].

### 3.2. Cancer

It has been demonstrated that tumor antigen-pulsed DCs primed MHC class I- and class II-restricted antigen specific T cells in vivo and stimulated the regression of established renal cell carcinoma and melanoma [68]. As previously mentioned, DCs may be important target cells for the development of a clinically applicable cancer vaccine. To date, several different types of cancer vaccines have been developed, including tumor-associated polypeptides (or proteins), tumor cell lysates, and irradiated autologous tumor cells. In most of these approaches, immunization with antigens needs to be combined with strong adjuvants or cytokines (IL-2, GM-CSF or IL-12) to induce a strong immune response. However, such strong adjuvants or cytokines are not used in humans because they usually elicit extensive unwanted side effects. Recently, it was found that administration of heat-killed tumor cells (hereafter referred to as HKs) into mice induced anti-tumor immune responses in the absence of adjuvants [69]. However, the underlying mechanism by which the HKs elicit strong anti-tumor immune responses has not yet been investigated. Thus, we took advantage of the IL-12 p40 reporter mice to investigate how HKs can induce potent anti-tumor immune responses. Immunization of mice with the HKs stimulates DCs and macrophages to induce IL-12 p40 expression (see Figure 9) and promotes expression of MHC and co-stimulatory molecules such as CD40 and CD86. Furthermore, we also found that HKs may promote migration of DCs to secondary lymphoid organs to facilitate antigen presentation to antigen-specific T cells by modulating the expression levels of chemokine receptors such as CCR5 and CCR7 (Gi *et al.*, manuscript in preparation).

**Figure 10.**
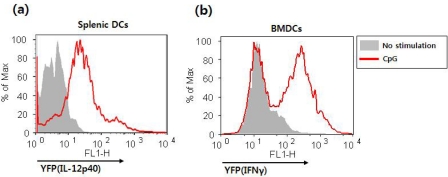
Detection of cytokine-producing NKDCs via IL-12 p40 or IFNγ reporter system. (a) Splenic DCs from IL-12 p40 reporter mice were isolated with MACS and subsequently stimulated with CpG for 16 hrs. On NK1.1^+^CD11c^+^ population gate, IL-12 p40 production was analyzed by flow cytometry and YFP expression. (b) BMDC from IFNγ reporter mice were expanded with IL-15. After 6 days, IFNγ production was analyzed by flow cytometry and YFP expression gated on NKDC populations.

Additionally, cytokine-producing NKDCs can be detected using the IL-12 p40 reporter system. Figure 10A shows that IL-12 production by splenic NKDCs upon stimulation with CpG can be measured by YFP expression. NKDC-like DC populations can be expanded by co-culture of BM cells with IL-15, and these cells have been known to produce IFNγ when stimulated with CpG. Figure 10B confirms the previous findings with BMDCs derived from IFNγ cytokine reporter mice [70]. Our studies illustrate that cytokine reporter mice can be very useful to examine potential anti-tumor reagents regarding DC activation as well as to define the characteristics of new DC subsets.

### 3.3. Allergies

Atopic dermatitis (AD), an allergic immune disease, is a chronic and relapsing inflammatory skin disease [71]. AD patients are usually hypersensitive to allergens and have high levels of IgE in their serum. In addition, immunological disturbances, such as overexpression of Th2 cytokines (e.g. IL-4, IL-5, IL-10) and downregulation of Th1 cytokines (e.g. IFNγ) have been noticed in patients with AD. However, the mechanisms of IgE hyperproduction and the pathogenesis of AD have not been clarified. Recently, inbred NC/Nga mice became available as an animal model for human AD [72]. Like human AD, NC/Nga mice have high levels of IgE in their serum, which is thought to result from a defective production of IFNγ from T cells. Matsumoto *et al.* have previously reported that atopic NC/Nga mice are incapable of IL-12 cytokine production [73]. To investigate whether IL-12 expression by DCs might be altered in NC/Nga mice, IL-12 p40 reporter alleles were introduced into the NC/Nga genetic background by backcrossing procedures. Using NC/Nga and C57BL/6 (B6) mice with the IL-12 p40 reporter, we compared IL-12 production by DC stimulation with TLR ligands such as CpG. Our data demonstrated that IL-12 producing DC populations in NC/Nga mice were reduced by about 25% compared to wild type B6 mice (data not shown). Thus, IL-12 p40 NC/Nga mice will be useful for monitoring DC activation and for identifying what type of innate immune cells produce IL-12 during the development of AD.

## 4. Conclusions

DCs play a critical role in determining the polarization of T cell immune responses, which have a direct impact on what types of antibody isotypes B cells produce. In other words, DCs can direct which adaptive immune responses are induced. Research on the functions of DCs should become a central focus of the immunology field because DCs are involved in various diseases, including infectious diseases, cancer, autoimmune diseases, and allergies.

Through recognition of appropriate danger signals, DCs help to protect the host from a variety of diseases and maintain a micro-environment for optimal immunity in healthy individuals. If DCs do not detect danger signals, the host immune system does not induce immune responses against pathogens and also may not induce immune tolerance upon induction of autoimmune responses. Considering the importance of DC functions in immunological regulation, DCs have been the focus for therapeutics for many diseases including cancers. Here, we have introduced the cytokine reporter mouse model as a tool for monitoring activation of DCs. To achieve better understanding of DC functions, it will be important to expand the studies of DC subsets in healthy and diseased states. To conclude, we anticipate a dramatic increase in the number of studies on DCs that will be based on populations that are isolated from mouse and human lymphoid organs or are cultured in vitro using DC growth factors.

## Figures and Tables

**Table 1.** Murine DC subsets.

**DC subsets**		**Features**			
		Site	Function	Phenotype	TLR expression
cDC	CD8α^+^	Lymphoid organ (T cell zone)	Cross presentation	CD11b^−^CD11c^+^	TLR1, 2, 4, 6, 7, 8, 9
	CD8α^−^	Lymphoid organ (Marginal zone)	APC	CD11b^hi^CD4^+^/^−^CD11c^+^	TLR1, 2, 3, 4, 6, 8, 9
pDC		Mast organs	APC	CD11b^−^B220^+^CD11c^+^	TLR1, 2, 4, 5, 6, 7, 8, 9
Langerhans cell		Skin	APC	CD11b^+^CD8α^−^CD4^+/−^CD11c^+^	TLR1, 3, 6, 7
Interstitial DC		Peripheral organ	APC	CD11b^+^CD8α^−^CD4^−^CD11c^+^	TLR1-8
NKDC		Lymphoid organ	Cytotoxicity/APC	NK1.1^+^B220^+^CD11c^+^	TLR9

**Table 2.** TLRs and their ligands.

**TLRs**	**Ligands**
TLR1/TLR2	Triacylated lipopeptides
TLR2/TLR6	Diacylated lipopeptides
TLR3	Viral dsRNA
TLR4	Lipopolysaccharide
TLR5	Flagellin
TLR7	Viral ssRNA
TLR8	Viral ssRNA
TLR9	Bacterial or viral CpG DNA
TLR11	Profilin
TLR10, 12, 13	Unknown
